# Analytical Sensitivity and Specificity of Two RT-qPCR Protocols for SARS-CoV-2 Detection Performed in an Automated Workflow

**DOI:** 10.3390/genes11101183

**Published:** 2020-10-12

**Authors:** Gustavo Barcelos Barra, Ticiane Henriques Santa Rita, Pedro Góes Mesquita, Rafael Henriques Jácomo, Lídia Freire Abdalla Nery

**Affiliations:** Research and development department, Sabin diagnostic medicine, Brasilia 70.632, Brazil; ticihenriques@gmail.com (T.H.S.R.); pedrogm@gmail.com (P.G.M.); rafaeljacomo@sabin.com.br (R.H.J.); lidia@sabin.com.br (L.F.A.N.)

**Keywords:** SARS-CoV-2, validation, RT-qPCR

## Abstract

WHO declared the novel coronavirus (COVID-19) outbreak a global pandemic on 11 March 2020. The establishment of standardized RT-qPCR protocols for respiratory secretions testing, as well as sharing of specimens, data, and information became critical. Here, we investigate the analytical performance of two interim RT-qPCR protocols (Charité and Centers for Disease Control (CDC)) for the qualitative detection of SARS-CoV-2 executed in a fully automated platform. Analytical specificity, PCR amplification efficiency, analytical sensitivity (limit of detection), and cross-reactivity were evaluated using contrived samples. The on-going accuracy was evaluated by retrospective analysis of our test results database (real clinical samples). N1, E, and a modified version of RdRP assays presented adequate analytical specificity, amplification efficiency, and analytical sensitivity using contrived samples. The three assays were applied to all individuals who requested the SARS-CoV-2 molecular test assay in our laboratory and it was observed that N1 gave more positive results than E, and E gave more positive results than RdRP (modified). The RdRP and E were removed from the test and its final version, based on N1 assay only, was applied to 30,699 Brazilian individuals (from 19 February 2020 to 8 May 2020). The aggregated test results available in the database were also presented.

## 1. Introduction

The World Health Organization (WHO) declared that the coronavirus disease 19 (COVID-19) outbreak constituted a Public Health Emergency of International Concern on 30 January 2020. The development of reliable laboratory diagnosis for severe acute respiratory syndrome coronavirus 2 (SARS-CoV-2) became mandatory to identify, isolate, and provide optimized care for patients early on [1]. On 11 March 2020, the WHO declared that the novel coronavirus (COVID-19) outbreak was a global pandemic [2] and the establishment of standardized RT-qPCR protocols for respiratory secretions testing, as well as sharing of specimens, data, and information became critical [3].

Two of the most popular RT-qPCR protocols to detect the SARS-CoV-2 were: (a) the assay originally proposed by the Charité-Universitätsmedizin Berlin Institute of Virology [4], and then endorsed by the World Health Organization [5]; (b) the assay developed by the Centers for Disease Control (CDC) [6]. The Charité protocol targets RNA sequences of E, RdRP, and N genes. The E gene assay is used as a screening tool because it detects all viruses from the *Sarbecovirus* subgenus (e.g., SARS-CoV, SARS-CoV-2, and related bat viruses) and then the RdRP gene assay is used as a confirmatory testing (specific to SARS-CoV-2). The N gene assay (also specific to SARS-CoV-2) can eventually be analyzed as an additional confirmatory assay [7]. The CDC protocol is comprised of three N gene assays (N1, N2, and N3), and all of them allow the specific detection of SARS-CoV-2 [8]. An additional primer/probe set for detecting human RNase P gene (RPP30) is included to check for the presence of human nucleic acids in the specimen [7]. 

Validation of these protocols is considered a key knowledge gap for COVID-19, especially if executed in a high-throughput automated format because false positive or false negative results can negatively impact not only the individual patient but also can have a broad public health impact [9]. During a pandemic, automated solutions for molecular diagnostics can help handle large numbers of samples intrinsic to the situation and quickly clear or confirm suspected cases [10]. So, we decided to use the Flow Flex Solution (Roche Diagnostics Ltd., Pleasanton, CA, USA) to perform the validation. This solution allowed us to test 93 samples and 3 controls in four hours and a half. 

Here, we investigate the analytical performance of these two interim RT-qPCR protocols (Charité and CDC) for the qualitative detection of SARS-CoV-2 executed in a fully automated platform. In addition, we present the stepwise evolution of the assay as positive samples were obtained in the daily routine and the test results database was analyzed. 

## 2. Materials and Methods 

### 2.1. Primary Samples Collection and Processing

Sixty nasopharyngeal swabs samples were collected from healthy volunteers using Rayon swab (*n* = 30) or cotton swabs (*n* = 30) and placed into cobas^®^ PCR Media tubes (Roche Molecular Systems, Inc. Basel, Switzerland) containing 4.3 mL of guanidine hydrochloride (<40%). Guanidine hydrochloride virtually inactivates all pathogens that may be present in the sample, preserves all nucleic acid of the specimen and protects the operator from contamination during sample manipulation [11,12,13,14]. Guanidine hydrochloride also guarantees the stability of the contrived samples, allowing us to introduce synthetic SARS-CoV-2 RNA into authentic nasopharyngeal specimens making the testing primary sample as real as possible. Before being processed, samples were vortexed, briefly spun, and the collection swab was removed from inside the tube and discarded by an operator protected by a laminar flow cabinet. Samples were then allocated into the Flow Flex Solution and the primary sample handling (PSH) transferred 200 μL of the primary tube content to an extraction cartridge. 

### 2.2. Nucleic Acid Extraction

Nucleic acids were extracted from 200 μL of the primary sample (present in the extraction cartridge) using the Flow Flex Solution associated MagNA Pure 96 Instrument and MagNA Pure 96 DNA and Viral NA Small Volume Kit (protocol Viral NA Universal version 4.0) with elution in 100 μL of elution buffer.

### 2.3. SARS-CoV-2 Assays Evaluated

SARS-CoV-2 RT-qPCR assay names adopted by Charité and the CDC were maintained to avoid mistakes: E, RdRP, N, N1, N2, and N3. Primers and probes sequences can be found in Table 1 (Charité) and Table 2 (CDC). Table 3 describes the primers and probes for RPP30, which check for the presence of human nucleic acid in the samples and our Artificial External “process” Control (AEC), an synthetic RNA that controls the entire process. All primers and probes were purchased from Integrated DNA technologies, Coralville, IA, USA. Note that probes are double quenched and contain internal quenchers (ZEN or TAO), which are not used by the original protocols.

### 2.4. One-Step RT-qPCR Reaction and Thermocycling Conditions

The RT-qPCR reaction (10 μL) consisted of 2 μL of LightCycler^®^ Multiplex RNA Virus Master (5×) (Roche, Basel, Switzerland), 0.75 μL (for N1, N2, N3, RdRP_Mod, and E_Mod) or 1 μL (for RdRP, E, and N) of SARS-CoV-2 primer/probe assay (FAM, 0.2 μL of RPP30 primer/probe assay (HEX), 0.33 μL of AEC primer/probe assay (CY5), 0.05 μL of reverse transcriptase (200×) (Roche), 5 μL of extracted nucleic acid, and 1.67 μL or 1.42 μL of nuclease-free water (all oligos were from Integrated DNA technologies, Coralville, IA, USA). The stock concentration of each primer/probe in the reaction can be found in Table 1, Table 2 and Table 3. Thermocycling conditions were: reverse transcription: 10 min at 50 °C; polymerase activation: 3 min at 94 °C; followed by 45 cycles of 15 s at 95 °C and 30 s at 55 °C. The instrument used was LightCycler 480 II controlled by the Flow Flex system. The master mix was prepared by an operator and distributed into a 384-well plate by the PCR set up unit (PSU). After master mix distribution, the PSU also distributed the samples. Each sample was processed in duplicate.

### 2.5. SARS-CoV-2 Diagnostic Synthetic Sequence Production

A synthetic dsDNA molecule (gblock—Integrated DNA technologies, Coralville, IA, USA), comprising of the concatenation of all six viral assay target sequences in the SARS-CoV-2 genome (Table 3), was in vitro transcribed into its RNA form. The nucleotide sequence can be retrieved from GenBank with the accession number MT458696. Upon arrival it was diluted, quantified using Qubit^®^ DNA HS (High Sensitivity) Assay Kits (Thermo Scientific™, Waltham, MA, USA) on Invitrogen Qubit^®^ 4 Fluorometer (Thermo Scientific™, Waltham, MA, USA) and ten nanograms were used as a template in the in vitro transcription reaction. The in vitro transcription was performed with TranscriptAid T7 High Yield Transcription Kit (Thermo Scientific™) in accordance with manufacturer′s instructions. The in vitro transcribed RNA (2 μL) was quantified using Qubit^®^ RNA HS (High Sensitivity) Assay Kits (Thermo Scientific™) on an Invitrogen Qubit^®^ 4 Fluorometer (Thermo Scientific™). Nanogram to copy number conversion was performed as instructed by the synthetic dsDNA manufacturer on (https://www.idtdna.com/pages/education/decoded/article/calculations-converting-from-nanograms-to-copy-number).

### 2.6. Artificial Process Control Production

A random DNA sequence of 200 bases (NCBI GenBank accession number MF374500.1) was generated by the tool available at http://www.faculty.ucr.edu/~mmaduro/random.htm. The sequence was not available in public databases according to BLAST (NCBI) and was purchased as gblock from Integrated DNA technologies, Coralville, IA, USA. Artificial process control RNA was produced and quantified as described in SARS-CoV-2 diagnostic synthetic sequence production. MagNA Pure 96 Instrument added it (10^5^ copies/extraction) to each sample during the nucleic acid extraction.

### 2.7. Amplification Efficiency

Amplification efficiencies of each primer/probe set were investigated by testing 10-fold dilution (1.48 × 10^8^ to 1.48 × 10^2^ copies/PCR) of the synthetic SARS-CoV-2 diagnostic RNA encompassing a copy number range, which would be considered low, medium, and high viral loads in clinical samples, followed by the evaluation of the standard curve parameters, specially the slope, from which the amplification efficiency and the coefficient of determination (*R*^2^) are derived. These analyses were performed using linear regression tools available in Graphpad Prism software version 6.0 (Graphpad, Inc., La Jolla, CA, USA).

### 2.8. Determining Limit of Detection of Assays

Limits of detection were calculated by the probit regression analysis of a 1:2 serial dilution (from 2.64 × 10^4^ to 4.04 × 10^−1^ copies/reaction) of the synthetic SARS-CoV-2 diagnostic RNA. Three samples corresponding to each dilution point were tested in duplicate on two different days (*n* = 12) and the assay response (detected or not detected) was measured. Applying the probit regression analysis to the data, a probability of detection versus concentration was returned. The target concentration at which the assay tested positive 95% of the time (Limit of detection—LOD) was estimated using Minitab version 19 (Minitab, LLC., Centre, PA, USA). Because of the repeatability and reproducibility introduced in the LOD experiment described above, (3 biological replicates tested as 2 technical replicates on 2 different days, *n* = 12) this experiment also evaluates these parameters.

### 2.9. Cross-Reaction

To investigate the assays′ cross-reactivity with other respiratory viruses, pools of positive samples used in our laboratory as internal quality controls for RT-qPCR assays were tested (Influenza A, H1N1, and H3N2, parainfluenza virus 3 and 4, rhinovirus, coronavirus 229, and coronavirus HKU). As an alternative, in-silico cross-reaction studies can be performed and cross reactivity is defined as homology greater than 80% between one primer/probe and any sequence present in the target microorganism [9].

### 2.10. On-Going Test Accuracy Evaluation and Analysis of SARS-CoV-2 Laboratory Test Results Database

From 19 February 2020 to 8 May 2020 our laboratory applied the proposed method to 30,699 Brazilian patients. The first case of SARS-CoV-2 was detected on 04 March 2020. It was the central-west region of Brazil′s case zero. Several positive cases were subsequently detected and the test results and Cq values were anonymously retrieved from the laboratory test results database. Positive and negative agreements between selected assays were evaluated on two occasions: a) after the detection of 23 positive samples, and b) after the detection of 75 positive samples. In addition, all of our cases (from 19 February 2020 to 8 May 2020) were anonymously retrieved from the laboratory test results database and presented according to their Brazilian State of origin as absolute and relative frequency to evaluate the positivity difference between states and try to figure out systematic bias.

### 2.11. Ethical Considerations

All volunteers agreed to participate, signed informed consent, and the internal use of these samples for diagnostic workflow optimization was according to the medical ethical rules of our institution. Personal data was anonymized irreversibly for the analysis of the SARS-CoV-2 laboratory test results database. 

## 3. Results

### 3.1. Analytical Specificity

**Figure 1:**
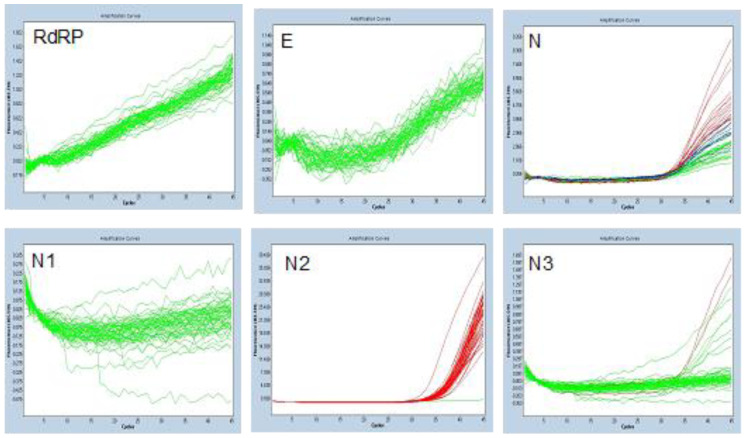
Assays’ analytical specificity analysis. N, N2, and N3 were excluded from the validation due to lack of analytical specificity when applied to nasopharyngeal samples of healthy volunteers.

The nasopharyngeal swabs samples from the 60 healthy volunteers were submitted to the six considered SARS-CoV-2 assays to check the generation of unspecific response. We observed consistent false-positive results for N and N2 (60 out of 60 samples for both). The N3 assay generated a false-positive signal or inconclusive results in 13 out of 60 tested samples. Because of this the N2, N3, and N assays were not considered for subsequent experiments due to the lack of analytical specificity under our conditions (Figure 1). This lack of analytical specificity was not observed for N1, E, and RdRP. In addition, E and RdRP assay primer/probe concentrations were modified (termed E (modified) and RdRP (modified)), and this concentration optimization did not interfere with the specificity of the assays (20 healthy volunteers’ samples tested—results not presented).

**Figure 2:**
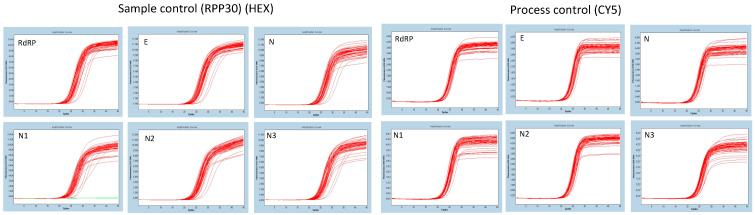
Figure depicting quality control targets. Left panel (sample control RPP30) and right panel—(process control AEC)—*n* = 60, 30 collected with Rayon swabs and 30 collected with cotton swabs. No difference was observed between both Rayon and cotton.

Rayon (*n* = 30) and cotton (*n* = 30) swabs were used for the nasopharyngeal sample collection for this first experiment. We did not observe any RT-PCR inhibition associated with cotton or Rayon by inspecting the RPP30 (HEX) and AEC (CY5) amplification curves (Figure 2).

### 3.2. Amplification Efficiencies

**Figure 3:**
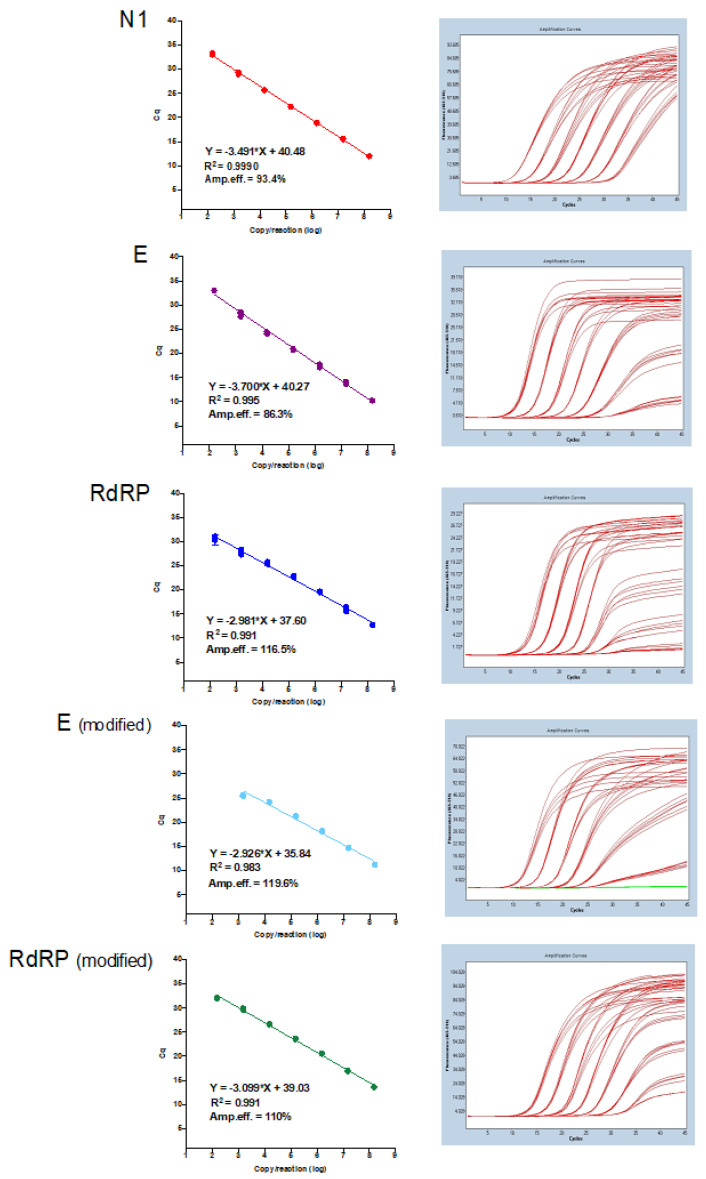
Assays′ amplification efficiency analysis. Left panel—linear regression analysis; right panel—raw data. The better amplification efficiency was observed for N1 (close to 100%), followed by RdRP (modified), E, RdRP, and E (modified).

Amplification efficiencies of N1, E, RdRP, E (modified), and RdRP (modified) assays were 93.4%, 86.3%, 116.5%, 119.6%, and 110%, respectively, when applied to serial dilution of 1:10 (1.48 × 10^8^ to 1.48 × 10^2^ copies/PCR) of the synthetic SARS-CoV-2 diagnostic RNA (Figure 3).

### 3.3. Limit of Detection

**Figure 4:**
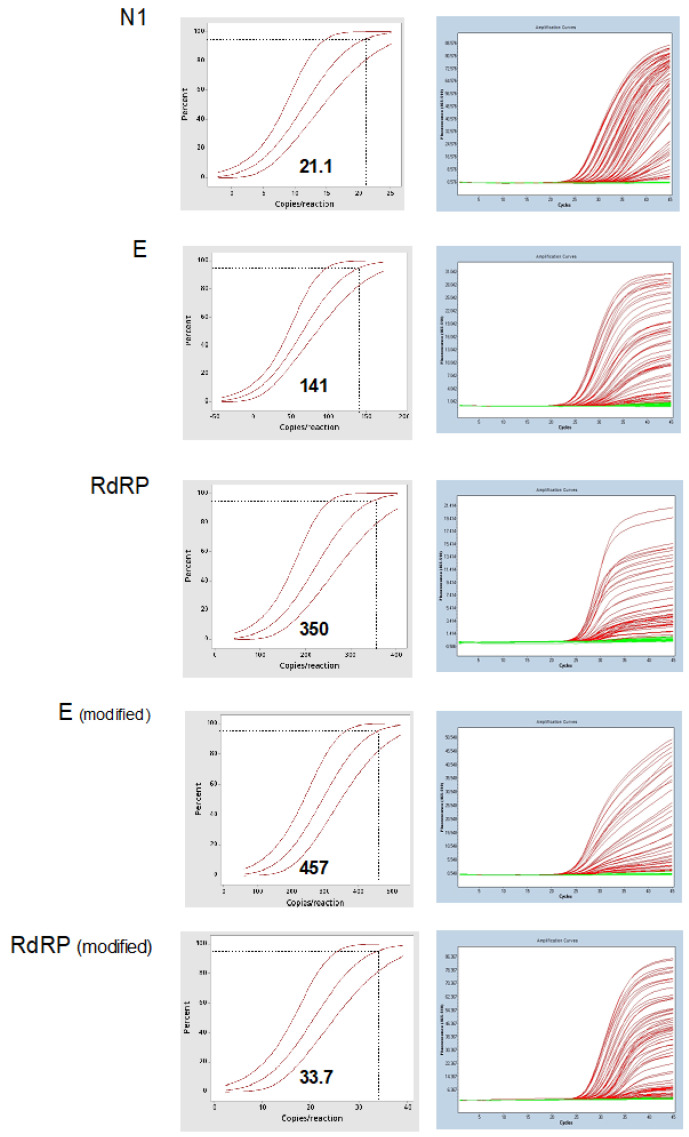
Determination of assays′ limit of detection. Left panel—probit regression analysis (inserted values unit are copies/reaction); right panel—raw data. N1 and RdRP (modified) showed better LOD, followed by E, RdRP, and E (modified).

Limiting dilution of 1:2 (from 2.64 × 10^4^ to 4.04 × 10^−1^ copies/reaction) of the synthetic SARS-CoV-2 diagnostic RNA was tested in order to evaluate the assays′ limit of detection. Probit regression analysis returned the limit of detection of 21 (95% CI 16.5–31.1) copies/reaction for N1, 141 (95% CI 109–207) copies/reaction for E, 350 (95% CI 281–508) copies/reaction for RdRP, 457 (95% CI 382–598) copies/reaction for E (modified), and 33.7 (95% CI 27.6–46.8) copies/reaction for RdRP (modified) (Figure 4).

### 3.4. Cross-Reaction

When applied to pools of positive samples used in our laboratory as internal quality controls for RT-qPCR assays for other respiratory viruses (influenza A, H1N1, and H3N2, parainfluenza virus 3 and 4, rhinovirus, coronavirus 229, and coronavirus HKU) N1, RdRP, RdRP (modified), E, and E (modified) assays did not return false-positive results. The in-silico cross-reactivity analysis of all primers used in this study was described elsewhere [4,15,16].

### 3.5. On-Going Test Accuracy Evaluation

**Figure 5:**
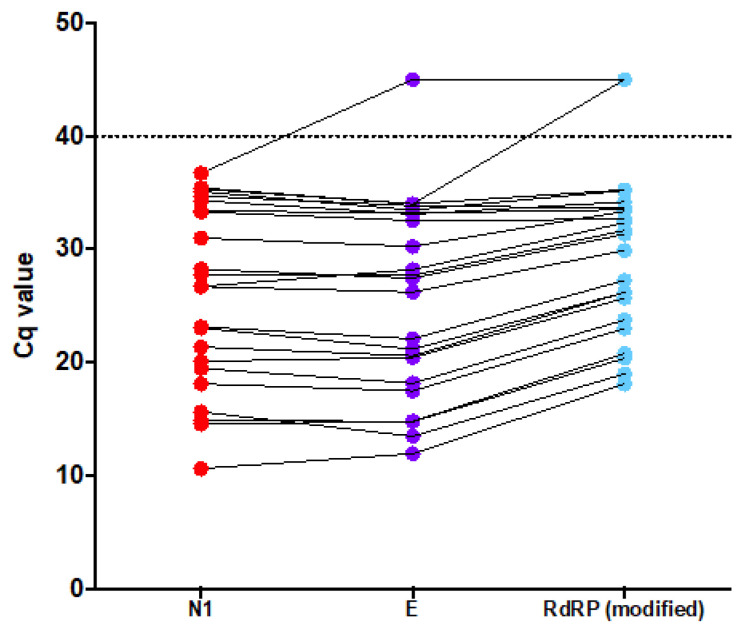
Cq values observed for the first 23 positive samples detected by the first version of the proposed method: N1 (red), E (magenta) and RdRP modified (R) (light green). N1 and E showed lower Cq values than RdRP (modified). Dashed line depicts the cut-off value of >40.

The first version of the proposed method was based on N1, E, and RdRP (modified) because these assays showed better amplification efficiencies and consequently better limits of detection in the experiments using contrived samples. The test started to be applied to the population of Brazil’s Federal District on 19 February 2020 and the first positive sample was detected on 4 March 2020 (Brazil’s Federal district’s case zero). After processing 968 samples (23 tested positive and 945 tested negative), it was noted that median (min–max) Cq values for N1 were 26.71 (10.61–36.72), for E were 27.44 (11.93–> 40), and for RdRP were 31.35 (10.61–> 40) (Cq > 40 meaning not detected) (Figure 5). Twenty-one samples returned positive and 942 results were negative for the three assays resulting in 91.3% positive agreement and 99.6% negative agreement. One sample was positive for N1 and E, but not for RdRP (modified). Another sample was positive only for N1. These resulted in 95.6% positive agreement and 99.7% negative agreement for N1 versus E. Hence, the RdRP assay was removed from the proposed method, which thereafter was based on N1 and E.

**Figure 6:**
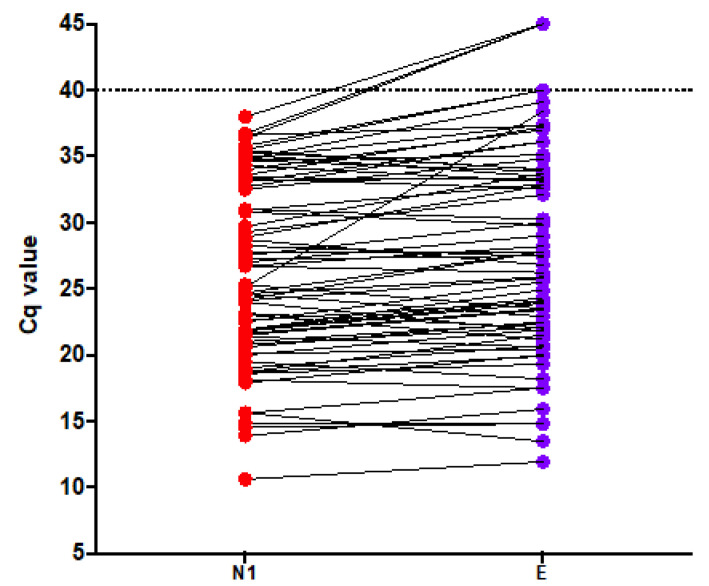
Cq values observed for the first 75 positive samples detected by the second version of the proposed method: N1 (red) and E (magenta). Dashed line depicts the cut-off value of >40. Three samples were positive only for N1 suggesting a better diagnostic capability for this assay.

After processing 2195 samples (75 positive and 2120 negative), it was noted that the median (min–max) Cq values for N1 were 25.20 (10.61–38.0) and for E were 27.12 (11.93–> 40) (Figure 6). Seventy-two samples returned positive results for both assays. Three samples were positive for N1, but not for E resulting in 96% positive agreement and 99.8% negative agreement. Hence, the E assay was removed from the proposed method, which thereafter was based on N1 only.

### 3.6. Analysis of SARS-CoV-2 Laboratory Test Results Database

From 19 February 2020 to 8 May 2020 our laboratory applied the proposed method to 30,699 Brazilian individuals (49.5% were women and 50.5% were men, and the average age was 42.5 ± 17,08 years for women and 41.7 ± 16.3 years for men) and 5596 (18.23%) tested positive. From this total of patients, 14,488 (47.1%) were from Brazil’s Federal District (where our core laboratory is located) and 1031 (7.12%) tested positive. The remaining 16,211 (52.8%) patients were from other Brazilian states (Table 4). Positivity was different between the states. States from Northern Brazil (e.g., Amazonas, Acre, Pará, Roraima, and Rondônia) showed the highest proportions of positive results. 

### 3.7. Results Summary

The summary of all results obtained in this study can be found in Table 5. 

## 4. Discussion

Here, the following analytical performance characteristics of Charité and CDC protocols for SARS-CoV-2 detection were evaluated: (a) analytical specificity, which refers to the qPCR assay detecting the appropriate target sequence rather than other nonspecific targets also present in a sample [17]; (b) PCR amplification efficiency, which is the increase in amplicon per cycle, and is highly dependent on the primers used [17,18]; (c) analytical sensitivity or limit of detection, which refers to the minimum number of nucleic acid copies in a sample that can be detected with 95% probability [17]; (d) cross-reactivity with other pathogens; (e) on-going accuracy in clinical specimens, which refers to agreement between the test method and another method during the daily routine. 

We observed in the analytical specificity experiment that 3 out of 6 tested assays (N2, N3, and N) presented unspecific amplification on known negative samples from healthy volunteers (SARS-CoV-2 had not arrived in Brazil at that time) and they were excluded from the validation. This result could be due to intrinsic primer non-specificity or contamination with viral templates during oligo manufacturing [19]. In addition, no amplification inhibition was observed when cotton swabs were used, so cotton can be used for sample collection during a Rayon swab contingency.

Next, contrived positive samples were created by spiking synthetic SARS-CoV-2 RNA into known negative nasopharyngeal specimens. To avoid degradation of the synthetic SARS-CoV-2 RNA by the RNAses of the samples, collection tubes containing guanidine hydrochloride were used. These contrived samples were used to evaluate the E, RdRP, N1, E modified, and RdRP modified assays’ amplification efficiency (modified assays have their originally proposed primer concentrations changed). N1, E, and RdRP (modified) showed the better (closer to 100%) amplification efficiencies (93.4%, 86%, and 110%, respectively), suggesting that they could have better diagnostic capability. 

In the limit of detection experiment, the N1 and RdRP (modified) showed the highest analytical sensitivity for their RNA targets, 21 and 33.7 copies/reaction, respectively. The E assay, in its original concentration, was considered a tertiary assay (141 copies/reaction). Taken together, N1, RdRP (optimized), and E presented appropriate analytical sensitivity and specificity in our automated RT-qPCR workflow for the COVID-19 virus. The E assay was at least 4-fold less sensitive than the others using contrived samples. The limits of detection observed in this study were slightly different than those described for RdRP (3.6 copies per reaction) and E (3.9 copies per reaction) in their original description, where the authors used the in-vitro transcribed SARS-CoV-2 RNA directly in the RT-qPCR reaction [4]. Our results could reflect the fact that we spiked the synthetic RNA in the nasopharyngeal samples, to resemble a real clinical sample and for the limit of detection calculation we assumed that the nucleic acid purification recovered 100% of the spiked RNA sequences of the 200 μL aliquot used in the extraction.

Cross-reaction was not observed for the tested organisms either in vitro or in silico (in silico data were not presented and can be found elsewhere [4,15]).

No commercial method for SARS-CoV-2 detection was available for comparison in our territory at the time of this assessment (late January 2020). Hence, until the finding of circulating SARS-CoV-2, this validation was considered an on-going validation because of the lack of the test accuracy evaluation. Once some positive cases were detected, a retrospective head-to-head comparison of N1, E, and RdRP (modified) was anonymously performed on the test results database. It was observed that N1 gave more positive results than E, and E gave more positive results than RdRP (modified). RdRP (modified) was removed from the daily routine after the comparison of the three assays on 23 positive samples, and then the E assay was removed from the assay after its comparison with N1 on 75 positive samples. 

The observation that E had better diagnostic capability than RdRP (modified) contradicts the findings using contrived samples, because RdRP (modified) showed better amplification efficiency and limit of detection than E. This contradiction could reflect differences in the target abundance in the sample due to the presence of cellular content. Upon cell entry, SARS-CoV-2 hijacks ~60% of the cell expression capability and its replication relies on the production of genomic RNA but also on the expression of several shorter subgenomic RNAs coding for conserved structural proteins—spike protein (S), envelope protein (E), membrane protein (M), and nucleocapsid protein (N)—important for assembling progeny virions [20]. RdRP is located on ORF1b and its expression requires ribosomal frameshifting, implying that it is produced at significantly lower levels compared to ORF1a-encoded functions and subgenomic RNA. So, in the cellular content of the sample, the RdRP copy number is lower than E and N1. On the other hand, the E and N1 target is highly expressed, especially the N gene, because its sequences are present in almost all subgenomic RNA [20,21,22]. This fact also corroborates with the better diagnostic capability of N1 compared to E. In conclusion, N1 has better LOD, and amplification efficiency, and its target sequencing is highly abundant in infected cells present in the sample.

Because the only *Sarbecovirus* that circulated in humans in 2020 was the SARS-CoV-2 and a false negative result can have broad public health impact we decided not to report indeterminate results. If N1, E, or RdRP (modified) returned positive amplification the sample was considered positive. This protocol was in accordance with the Pan American Health Organization guidelines [23]. In addition, decreasing the number of assays executed for each sample to reach a diagnosis did not affect the test performance and was in accordance with the resource saving priority during a pandemic to avoid the collapse of test availability. 

More than 30,000 individuals were tested by the proposed method in almost 3 months and 18% tested positive. States from Northern Brazil presented higher levels of positive rates. Because epidemic stages between Brazilian states were different due to the size of the country, different levels of positivity between them is an ecologic evaluation of the assay, as similar proportions of positive results would indicate a systematic bias in the assay’s performance.

In conclusion, this study highlights the importance of local validation of in-house assays before their availability to the population. Experiments to establish the assay’s analytical specificity and sensitivity can be easily implemented. The proposed method detected the first case of SARS-CoV-2 in the central west region of Brazil, demonstrating that the use of the synthetic RT-qPCR target to investigate a novel assay’s diagnostic parameters in automated workflows is a quick, simple and effective way to be prepared for upcoming threats. The use of spiked samples that resemble real clinical specimens exposes the artificial SARS-CoV-2 sequences to the same background of nucleic acids yields that can be found in the routine and similar amplification behavior of real SARS-CoV-2. However, differences in the E and RdRP assays’ performance between contrived and real samples were observed and a retrospective on-going accuracy evaluation on our test database was necessary to make the assay reach its most reliable configuration. This observation indicates that the use of real positive samples is also essential for RT-qPCR assay validation.

## Figures and Tables

**Table 1:** RT-PCR primer and hydrolysis probe sequences, according to Corman et al., 2020 [4].

Assay	Name	Sequence (5′–3′)	Stock Solution
E	Primer F	ACAGGTACGTTAATAGTTAATAGCGT	4 μM (E) or 15 μM (E modified)
E	Primer R	ATATTGCAGCAGTACGCACACA	4 μM (E) or 15 μM (E modified)
E	Probe	FAM-ACACTAGCC/ZEN/ATCCTTACTGCGCTTCG-ABkFQ	2 μM (E) or 3.75 μM (E modified)
RdRP	Primer F	GTGARATGGTCATGTGTGGCGG	6 μM (RdRP) or 15 μM (RdRP modified)
RdRP	Primer R	CARATGTTAAASACACTATTAGCATA	8 μM (RdRP) or 1 5 μM (RdRP modified)
RdRP	Probe 1	FAM-CCAGGTGGW/ZEN/ACRTCATCMGGTGATGC-ABkFQ	1 μM (RdRP) or 3.75 μM (RdRP modified)
RdRP	Probe 2	FAM-CAGGTGGAA/ZEN/CCTCATCAGGAGATGC-ABkFQ	1 μM (RdRP) or 3.75 μM (RdRP modified)
N	Primer F	CACATTGGCACCCGCAATC	6 μM
N	Primer R	GAGGAACGAGAAGAGGCTTG	8 μM
N	Probe	FAM-ACTTCCTCA/ZEN/AGGAACAACATTGCCA-ABkFQ	2 μM

Legend: W is A/T; R is G/A; M is A/C.

**Table 2:** RT-PCR primer and hydrolysis probe sequences, according to the Centers for Disease Control (CDC) [6].

Assay	Name	Sequence (5′–3′)	Stock Solution
N1	Primer F	GACCCCAAAATCAGCGAAAT	15 μM
N1	Primer R	TCTGGTTACTGCCAGTTGAATCTG	15 μM
N1	Probe	FAM-ACCCCGCAT/ZEN/TACGTTTGGTGGACC-ABkFQ	3.75 μM
N2	Primer F	TTACAAACATTGGCCGCAAA	15 μM
N2	Primer R	GCGCGACATTCCGAAGAA	15 μM
N2	Probe	FAM-ACAATTTGC/ZEN/CCCCAGCGCTTCAG-ABkFQ	3.75 μM
N3	Primer F	GGGAGCCTTGAATACACCAAAA	15 μM
N3	Primer R	TGTAGCACGATTGCAGCATTG	15 μM
N3	Probe	FAM-AYCACATTG/ZEN/GCACCCGCAATCCTG-ABkFQ	3.75 μM

Legend: Y is C/T.

**Table 3:** RT-PCR primer and hydrolysis probe sequences for the sample collection control (RPP30) [6] and for the artificial external RNA control (AEC) (This study).

Assay	Name	Sequence (5′–3′)	Stock Solution
RPP30	Primer F	AGATTTGGACCTGCGAGCG	4 μM
RPP30	Primer R	GAGCGGCTGTCTCCACAAGT	4 μM
RPP30	Probe	HEX-TTCTGACCT/ZEN/GAAGGCTCTGCGCG-ABkFQ	2 μM
AEC	Primer F	GGGACTTTAAGCCGAGTCAAT	4 μM
AEC	Primer R	TGGTGGATCACAGTTTGTCAG	4 μM
AEC	Probe	Cy5-ACAGAGTTT/TAO/ACCGCATCTTGCCGT-IAbRQSp	2 μM

**Table 4:** Case distribution by state.

State	Total (*n*)	Positive (*n* and %)
Acre	3	1 (33.3%)
Amazonas	5964	2811 (47.1%)
Bahia	1162	102 (8.7%)
Ceará	1	0
Distrito Federal	14488	1031 (7.1%)
Espirito Santo	1	0
Goiás	635	58 (9.1%)
Maranhão	1	0
Minas Gerais	1459	134 (9.1%)
Mato Grosso do Sul	374	33 (8.8%)
Mato Grosso	672	35 (5.2%)
Pará	1156	790 (68.3%)
Paraná	806	70 (8.6%)
Rio de Janeiro	15	3 (20%)
Rio Grande do Norte	5	1 (20%)
Rondônia	2	1 (50%)
Roraima	131	18 (13.7%)
Rio Grande do Sul	2	0 (0)
Santa Catarina	931	110 (11.8%)
Sergipe	1	0 (0)
São Paulo	2665	382 (14.3%)
Tocantins	225	16 (7.1%)

**Table 5:** Performance characteristics of two RT-qPCR protocols for SARS-CoV-2 detection performed in an automated workflow.

Assay	Analytical Specificity (False-Positives)	Amplification Efficiencies (%)	Limit of Detection (Copies/Reaction—(95% CI )	Cross Reaction	Positive Agreement with N1	Negative Agreement with N1
E (Charité)	0 out of 60	86.3	141 (109–207)	No	72/75 (96%)	2117/2120 (99.8%)
RdRP (Charité)	0 out of 60	116.5	350 (281–508)	No	Excluded #	Excluded #
N (Charité)	60 out of 60	Excluded *	Excluded *	Excluded *	Excluded *	Excluded *
N1 (cdc)	0 out of 60	93.4	21 (16.5–31.1)	No	Reference	Reference
N2 (cdc)	60 out of 60	Excluded *	Excluded *	Excluded *	Excluded *	Excluded *
N3 (cdc)	13 out of 60	Excluded *	Excluded *	Excluded *	Excluded *	Excluded *
E_Modified	0 out of 60	119.6	457 (382–598)	No	Excluded #	Excluded #
RDRP_modified	0 out of 60	110	33.7 (27.6–46.8)	No	21/23 (91.3%)	942/945 (99.6%)

* Lack of analytical specificity; # lack of analytical sensitivity.
